# Junior Doctor Mentors: Enhancing Medical Student Psychiatry Training

**DOI:** 10.1192/bjo.2022.148

**Published:** 2022-06-20

**Authors:** Kenneth Ruddock, Catriona Neil

**Affiliations:** NHS Lanarkshire, Lanarkshire, United Kingdom

## Abstract

**Aims:**

Increased clinical contact during undergraduate psychiatry placements has been shown to both increase the likelihood of students considering a career in psychiatry and reduce mental-health related stigma. It can be challenging to provide medical students with a valuable clinical experience, a problem which has been further exacerbated by the coronavirus pandemic. We aimed to develop a junior doctor mentoring scheme to increase clinical exposure and teaching for students.

**Methods:**

Within NHS Lanarkshire, groups of 4–6 University of Glasgow medical students are accommodated for their five-week clinical psychiatry blocks, during which they have a varied structured timetable, providing an overview of different psychiatric specialties. As such, students meet a wide range of clinicians, which can unfortunately mean there is little continuity in their training throughout the block.

We developed a mentoring scheme to help address this issue. Enthusiastic trainee doctors – including foundation year two doctors, GP trainees and psychiatry core trainees – were invited to participate. Medical students are paired with a mentor for the duration of their block, during which they meet informally on a weekly basis. Mentors provide students with ward shadowing opportunities and bedside teaching, as well as completing work-place based assessments (WPBAs), discussing case reports and providing an additional perspective for end-of-block reports.

**Results:**

The mentoring scheme has been running successfully for every five-week student placement since October 2020. Student feedback has been collected via an anonymous electronic questionnaire. Students were asked what they enjoyed the most about their placement, with students frequently highlighting the support from their mentor.

Examples from free-text comments included, “having an assigned mentor was really useful as someone to touch base with and go through clinical cases” and, “having a mentor was invaluable – it is crucial to have a friendly face on the wards and a contact to complete WPBAs”.

Informal feedback from mentors has also been positive with trainees enjoying the opportunity to develop their teaching skills and support student training. Mentors also highlighted the role's benefit for their portfolios and specialty applications.

**Conclusion:**

This simple and cost-free intervention has had resoundingly positive feedback from medical students and trainees. Medical students enjoy having consistent informal teaching, support and feedback. Our mentoring scheme will continue for all medical students in NHS Lanarkshire and we would encourage other areas to consider a similar project. By increasing clinical exposure we hope to further reduce mental health stigma amongst students and inspire the psychiatrists of tomorrow.

